# IS CALF CIRCUMFERENCE ASSOCIATED WITH CLINICAL AND NUTRITIONAL OUTCOME IN OLDER PATIENTS?

**DOI:** 10.1590/0102-672020230055e1773

**Published:** 2023-11-13

**Authors:** Lucas Rosasco MAZZINI, José Luis Braga de AQUINO, José Gonzaga Teixeira de CAMARGO, Vania Aparecida LEANDRO-MERHI

**Affiliations:** 1 Pontificia Universidade Católica de Campinas, School of Medicine – Campinas (SP), Brazil.; 2 Pontificia Universidade Católica de Campinas, Postgraduate Program in Health Sciences – Campinas (SP), Brazil.; 3 Pontificia Universidade Católica de Campinas, School of Life Sciences – Campinas (SP), Brazil.

**Keywords:** Nutritional status, Anthropometry, Inpatients, Aged, Estado nutricional, Antropometria, Pacientes internados, Idoso

## Abstract

**BACKGROUND:**

Previous studies have shown a relationship between calf circumference (CC) and outcomes in hospitalized patients.

**AIMS:**

To investigate the relationship between CC and clinical and nutritional outcomes in older in-patients (OiP) in a surgery ward.

**METHODS:**

This was a cross-sectional study with 417 OiP in a surgery ward. Clinical variables, anthropometry, and nutritional screening instruments such as subjective global assessment (SGA), mini nutritional assessment (MNA), and nutritional risk screening (NRS) were used in the investigation. The tests Pearson’s chi-square, Mann-Whitney, Kruskal-Wallis, and Spearman’s coefficient, and multiple linear regression analyses were used to review the factors associated with CC.

**RESULTS:**

Lower CC values were found in the age group 80 years and over (p<0.0001), presence of complications (p=0.0269), NRS (p<0.0001), SGA (p<0.0001), and MNA (p<0.0001). Gender (p=0.0011; partial R^2^=0.01151), age (p=0.0002; partial R^2^=0.06032), body mass index (p≤0.0001; partial R^2^=0.40820), and arm circumference (p≤0.0001; partial R^2^=0.11890) are variables that together were associated with CC. There was also a relationship between SGA (p=0.0166; partial R^2^=0.00605) and absence of complications during hospitalization (p=0.0047; R^2^=0.01154) with CC.

**CONCLUSIONS:**

Gender, age, body mass index, and arm circumference were jointly associated with CC, in addition to SGA and absence of complications. The CC is a relevant indicator for OiP in the clinical practice.

## RESUMO

**RACIONAL:**

Estudos anteriores mostraram uma relação entre a circunferência da panturrilha (CP) e desfechos em pacientes hospitalizados.

**OBJETIVOS:**

Investigar a relação entre CP e desfecho clínico e nutricional em pacientes idosos hospitalizados (PIH) em uma enfermaria cirúrgica.

**MÉTODOS:**

Este foi um estudo transversal com 417 PIH em uma enfermaria de cirurgia. Variáveis clínicas, antropometria e instrumentos de triagem nutricional como avaliação subjetiva global (ASG), mini avaliação nutricional (MAN) e triagem de risco nutricional (NRS) foram utilizados na investigação. Qui-quadrado, Mann-Whitney, Kruskal-Wallis, coeficiente de Spearman e análise de regressão linear múltipla foram usados para analisar os fatores associados a CP.

**RESULTADOS:**

Menores valores de CP foram encontrados na faixa etária de 80 anos (p<0,0001), complicações (p=0,0269), risco nutricional pelo NRS (p<0,0001), ASG (p<0,0001) e MAN (p<0,0001). Sexo (p=0,0011; R2 parcial=0,01151), idade (p=0,0002; R2 parcial=0,06032), IMC (p≤0,0001; R2 parcial=0,40820) e circunferência do braço (CB) (p≤0,0001; R2 parcial=0,11890) foram variáveis que juntas estiveram associadas com a CP. Também houve relação entre ASG (p=0,0166; R2 parcial=0,00605) e ausência de complicações durante a internação (p=0,0047; R2= 0,01154) com a CP.

**CONCLUSÕES:**

Sexo, idade, IMC e CB associaram-se conjuntamente com CP, ASG e ausência de complicações. CP é um indicador relevante em PIH na prática clínica.

## INTRODUCTION

According to the World Health Organization (WHO)^35^, the older adult population is growing rapidly worldwide and should reach more than 1.2 billion people in the year 2025. This growth could lead to increased demand for hospitals and health services in general. For this reason, the nutritional diagnosis and therapy of this population continue to be the focus of researchers’ attention, such as the investigation of nutritional indicators for use in older in-patients (OiP), alone and in combination^1,10,13,19,22^.

The vast use of nutritional screening instruments and anthropometry indicators that are routinely used in the hospital setting is already highlighted in the literature, especially when dealing with the evaluation of nutritional status in older patients^6,17,22,25,28,30,39^. Just as it is already known that there is no defined gold standard for the assessment of OiP, many studies have compared different instruments for the actual nutritional diagnosis of this population^6,10,28,30,39^.

Analyzing the results and applicability of these nutritional evaluation methods in OiP, recent evidences^2,12^ showed that the nutritional risk screening (NRS) and the mini-nutritional assessment (MNA) moderately agreed in the assessment of older adults^2^, and the NRS was considered a predictor of clinical outcomes^12^. Other studies have shown the estimation of calf circumference (CC) cutoff points as a screening tool for reduced muscle mass^18^.

Although the use of CC is not a new indicator, its application has been investigated particularly in OiP, as demonstrated in recent publications^4,11,27^. If we take into account that many OiP are bedridden, unable to walk, and therefore, unable to assess their body weight, this indicator could become a valuable method for identifying the nutritional status of these patients.

The objective of this study was to investigate the relationship between CC and clinical and nutritional outcomes in OiPs in a surgery ward.

## METHODS

### Study design

This study was carried out in a university hospital that serves a representative population of adult and older adult patients in general, in a large metropolitan region. This was a cross-sectional and retrospective study, originally based on the selection of a population of approximately 500 OiP due to different diseases, admitted to a surgical ward.

The inclusion criteria for the participation were patients aged 65 years or older, who had undergone nutritional assessment procedure within the first 24 hours of admission, with diseases other than terminal, and with complete medical records in the institution. Patients hospitalized only for diagnostic investigation, those with dementia, and who remained in the hospital only for a period of less than 24 hours were excluded. After reviewing the inclusion and exclusion criteria, a total of 417 patients met the eligibility criteria for participation to be started within 24 hours after admission. The study was initiated after approval by the institution’s Ethics and Research Committee (reference number: nº 3.587.982, CAAE 150277 19.0.0000.5481).

### Methodological procedures and variables assessed

Data collection took place through a search in the medical records, assessing the variables gender, age, type of disease, comorbidities, surgery, complications, lymphocyte count^29^, and length of hospital stay. Next, all registries of nutritional status evaluation were collected from the medical records, such as anthropometry indicators and the instruments for screening and assessment.

Anthropometry indicators included body mass index (BMI) (*underweight, adequate or normal weight, and overweight*) classified as recommended by Lipschitz^23^ for older people; the cutoff point of 22 kg/m^2^ identified patients with low body weight. Arm circumference (AC), triceps skinfold (TS), and arm muscle circumference (AMC) were evaluated according to previously defined criteria^5,15^ and CC was classified according to the WHO^36^.

For the nutritional screening and assessment instruments, the nutritional status was classified by the subjective global assessment (SGA)^9^ (*well nourished and malnourished*), and by the MNA^16^ (*eutrophic, risk of malnutrition, and malnourished*), and the nutritional risk was classified by the NRS^20^ (*with nutritional risk and without nutritional risk*).

### Statistical analysis

To describe the characteristics of the population assessed, frequency tables were developed for categorical variables with absolute frequency (n) and percentage (%) values, and for quantitative variables, descriptive measures were obtained as mean, standard deviation, and median. To compare proportions, the Pearson’s chi-square test or the Fisher’s exact test was used, when necessary. For the comparison of continuous measures between two groups, the Mann-Whitney test was applied, and among three or more groups, the Kruskal-Wallis test. To verify the relationship between numerical variables, Spearman’s linear correlation coefficient was used. To review the factors associated with CC, multiple linear regression analysis was adopted with the stepwise criterion for selecting variables. Multiple linear regression was operated aimed at identifying the variables associated with CC, but not to predict this result, by the assumption of cause and effect. Due to the transformation applied, the estimated parameters only served to direct the existing relationship and not for the calculation of predicted values. The transformation by ranks was chosen due to the absence of normality of the variables. The significance level adopted for the statistical tests was 5%^7,14,32^.

## RESULTS

A population of 417 OiP was investigated, of which 64.3% (n=268) were male and 35.7% (n=149) were female. A total of 73.9% (n=308) was between 65 and 79 years of age and 26.1% (n=109) were 80 years and over. The rates of malnutrition by the instruments and the assessed indicators were different among them: 48.4% (n=202) were at nutritional risk according to the NRS; 14.6% (n=61) with malnutrition by MNA; 49.9% (n=208) at risk of malnutrition by MNA; 23.7% (n=318) with malnutrition according to SGA; and 26.1% (n=100) were underweight according to the BMI; the CC was 32.49±4.36 cm (Tables 1 and 2).

**Table 1:** General characteristics of the studied population (n=417).

Variables	Category	n	%
Age group (years)	65–79	308	73.9
	≥80	109	26.1
Gender	Female	149	35.7
	Male	268	64.3
Reason for hospitalization	Digestive tract disease	52	12.5
	Kidney disease	42	10.1
	Respiratory disease	19	4.6
	Vascular disease	68	16.3
	Oncological digestive disease	151	36.2
	Orthopedic disease	28	6.7
	Thoracic disease	57	13.7
Body mass index	Overweight	131	34.2
	Adequate	152	39.7
	Low weight	100	26.1
Comorbidity	Yes	309	98.1
	No	6	1.9
Mini nutritional assessment	Malnourished	61	14.6
	Eutrophic	148	35.5
	Malnutrition risk	208	49.9
Subjective global assessment	Well nourished	318	76.3
	Malnourished	99	23.7
Nutritional risk screening	At risk	202	48.4
	Without risk	215	51.6
Death	No	401	96.2
	Yes	16	3.8
Complications	No	357	85.6
	Yes	60	14.4
Surgery	No	137	32.9
	Yes	280	67.1

**Table 2:** General characteristics of the studied population (n=417).

Variables	Measure	Mean±SD	Median
Age	years	73.99±6.92	72.0
Length of hospital stay	days	8.94±8.11	6.0
Body mass index	kg/m²	25.28±5.19	24.90
Arm circumference	cm	27.77±4.41	27.50
Calf circumference	cm	32.49±4.36	32.10
Triceps skinfold	mm	16.70±8.15	15.00
Lymphocytes	cel/mm³	1,519.33±948.70	1,355.00
Arm muscle circumference	cm	225.72±34.52	224.35

SD: standard deviation.

**Figure 1:**
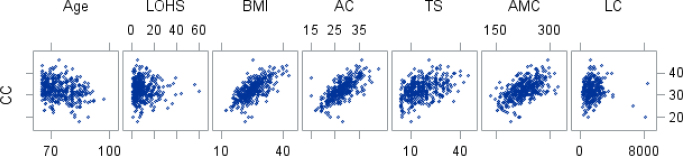
Dispersion between calf circumference measurements and study variables.

CC: calf circumference; LOHS: length of hospital stay; BMI: body mass index; AC: arm circumference; TS: triceps skinfold; AMC: arm muscle circumference; LC: lymphocyte coun.

Table 3 shows the correlation analysis results between CC and clinical and anthropometric variables, analyzed using Spearman’s correlation coefficient. There was a significant correlation between CC and age in an inverse and weak way. A direct moderate to high-intensity relationship was also observed with BMI, AC, TS, and AMC (Table 3 and Figure 1).

**Table 3:** Spearman’s correlation coefficient of calf circumference and clinical and anthropometric variables.

Variables	Coefficient (r)*	p-value^†^
Calf circumference *vs* age	-0.24848	<0.0001
Calf circumference *vs* length of hospital stay	-0.02028	0.6797
Calf circumference *vs* body mass index	0.66943	<0.0001
Calf circumference *vs* arm circumference	0.70746	<0.0001
Calf circumference *vs* triceps skinfold	0.38119	<0.0001
Calf circumference *vs* arm muscle circumference	0.59757	<0.0001
Calf circumference *vs* lymphocytes	0.10103	0.0827

* Spearman correlation coefficient; ^†^p<0.05.

There was a significant difference for CC between age groups (p<0.0001), presence of complications (p=0.0269), SGA (p<0.0001), NRS (p<0.0001), and MNA (p<0.0001). No difference was observed between gender (p=0.0689), type of disease (p=0.4076), surgery (p=0.5406), and death (p=0.4377) (Table 4). Lower values in CC mean or median were found in the group aged 80 years and over with clinical or surgical complications, at risk of malnutrition, or with malnutrition according to the NRS, SGA, and MNA.

**Table 4:** Descriptive analysis and comparison of calf circumference with the variables studied.

Variables	Mean ± SD	Median	p-value
Age group (years)			
65–79 (n=308)	33.13±4.19	33.00	<0.0001*
≥80 (n=109)	30.69±4.35	30.50	
Total (n=417)	32.49±4.36	32.10	
Gender			
Female (n=149)	31.97±4.73	32.00	0.0689*
Male (n=268)	32.78±4.12	32.50	
Total (n=417)	32.49±4.36	32.10	
Diseases			
Digestive (n=52)	33.43±5.04	32.15	0.4076^†^
Renal (n=42)	32.66±4.21	32.75	
Respiratory (n=19)	30.45±4.10	31.50	
Vascular (n=68)	32.27±4.45	32.25	
Digestive neoplasms (n=151)	32.32±4.37	32.50	
Orthopedics (n=28)	32.79±4.70	33.50	
Thoracic (n=57)	32.78±3.42	32.00	
Surgery			
No (n=137)	32.37±4.14	32.00	0.5406*
Yes (n=280)	32.56±4.47	32.50	
Complications			
No (n=357)	32.66±4.45	33.00	0.0269*
Yes (n=60)	31.47±3.66	31.50	
SGA			
Well nourished (n=318)	33.15±4.10	33.00	<0.0001*
Malnourished (n=99)	30.38±4.52	30.00	
NRS			
At risk (n=202)	31.21±4.36	31.00	<0.0001*
Without risk (n=215)	33.70±4.01	34.00	
MNA			
M+MR (n=269)	31.49±4.44	31.00	<0.0001*
Eutrophic (n=148)	34.32±3.56	34.00	
Death			
No (n=401)	32.52±4.36	32.30	0.4377*
Yes (n=16)	31.83±4.40	30.50	

* Mann-Whitney; ^†^Kruskal Wallis. SGA: subjective global assessment; NRS: nutritional risk screening; MNA: mini nutritional assessment; M+MR: malnutrition plus malnutrition risk.

Table 5 shows the results of the multiple linear regression analysis of the study factors associated with CC. It was found that gender (p=0.0011; partial R^2^=0.01151), age (p=0.0002; partial R^2^=0.06032), BMI (p≤0.0001; partial R^2^=0.40820), and AC (p≤0.0001; partial R^2^=0.11890) are variables that together were associated with the CC measurement. There was also a relationship between the classification of nutritional status by SGA (p=0.0166; partial R^2^=0.00605) and the absence of complications during hospitalization (p=0.0047; R^2^=0.01154) with the CC measurement (Table 5).

**Table 5:** Results of multiple linear regression analysis for the study of factors associated with calf circumference.

Variable	Estimated parameter*	p-value	Partial R^2^	
Age	-0.13231	0.0002	0.06032	age ¯calf circumference
Body mass index	0.27155	<0.0001	0.40820	BMI calf circumference
Arm circumference	0.52190	<0.0001	0.11890	AC calf circumference
Gender (M *vs* F)	27.20215	0.0011	0.01151	male calf circumference
Complications (no *vs* yes)	32.64815	0.0047	0.01154	uncomplicated calf circumference
Subjective global assessment (well-nourished *vs* malnourished)	23.41684	0.0166	0.00605	Well-nourished calf circumference

R^2^ model = 0.6165. *Estimated value; R^2^: coefficient of determination: partial; R^2^: proportion of response variability explained exclusively by the variable in question; R^2^ model: proportion of explanation of the dependent variable by the variation of the independent variables that remained in the model. Stepwise criterion used for selecting variables; AC: arm circumference; BMI: body mass index; M: male; F: female.

## DISCUSSION

The male gender and neoplastic diseases were prevalent in this investigation involving OiP. Although most older adult patients were aged between 65 and 79 years, there was a significant proportion of older patients aged 80 years and over. The CC mean or median values found herein are within the normal range, according to the adopted reference standard^34^.

As observed in other studies^6,10,30,39,40^, different rates of malnutrition were verified by comparing the indicators and the nutritional assessment instruments used in the population examined^31^. The NRS was a sensitive tool for diagnosing patients at nutritional risk (48.4%); by clustering the malnourished patients with those at risk of malnutrition, according to the MNA, the total rate was 64.5%. These two instruments have been considered valid methods in the assessment of OiP^2,6,12^.

Due to the variability of the different values of malnutrition found in the assessment of OiP, several reports considered the use of many indicators and tools for nutritional diagnosis^24^. This issue was observed in a recent prospective study that used the same nutritional screening and anthropometry instruments, adopted in the present study, to investigate the nutritional status of older adults and the predictive value of those instruments in the mortality of OiP^38^. The study in question identified that both NRS and MNA could predict mortality, but only NRS was the independent predictor of mortality^37^. Another recent article also showed that only NRS was an independent predictor of unfavorable clinical outcomes in OiP^12^. The CC investigation and its association with mortality was also investigated in a recent systematic review and meta-analysis, showing that low levels of CC were associated with a higher risk of mortality^33,36^; such observations also point to the relevance of using the CC indicator in hospital clinical practice. Another important aspect is the presence of wasting syndrome and its associated factors in OiP, as observed in another work that indicated a high prevalence of this syndrome associated with clinical, biochemical, and nutritional variables, including CC^11^. In general, all anthropometric nutritional indicators can be used to assess nutritional risk in older adult patients^3^.

Our results revealed a significant relationship of moderate to high intensity between CC and other anthropometric indicators, which could suggest the use of CC to replace the other indicators for assessing the nutritional status in OiP. Another relevant finding of our study was the association between CC and age group, presence of complications, SGA, NRS, and MNA. It is important to emphasize herein that in a work that investigated the CC, laboratory tests, and NRS, CC was considered a simple, non-invasive method and an effective measure to predict the nutritional risk in hospitalized patients over 80 years of age^38^.

Our study found no association between CC and diseases. Other papers have pointed to CC as an easy measure capable of predicting falls in older adult patients on hemodialysis^27^, and as a valuable tool for predicting the risk of sarcopenia in OiP with hip fractures^4^; also associated low CC values with frailty in diabetics older adult aged over 80 years^40^, and that CC showed good accuracy, sensitivity, and specificity for detecting malnutrition in both genders^3^. Another recent study that investigated the relationship between CC and AMC demonstrated that CC was associated with the risk of pneumonia^26^.

Our investigation showed that CC is an effective and easily applicable measure in the hospital setting. The most relevant findings of our investigation were the data observed by the multiple linear regression analysis for the study of factors associated with CC, in which it was possible to verify that the variables gender, age, BMI, and AC taken together were associated with CC and SGA and with the absence of complications during hospitalization. With these findings, it is possible to indirectly infer that this CC indicator may be better able to assess the nutritional status of OiP. The findings in our study are in line with other recent papers pointing to the relevance of using CC in older adults instead of other methods, especially if we consider those frequent situations of patients who are completely bedridden, unable to walk, and unable to measure body weight^21,36^. This study suggests even greater attention by healthcare professionals in treating the risk of sarcopenia in older adult patients, a situation already reported in the literature^8^. Under those conditions, CC indicator would be a very valuable method.

The strengths of the present study included the proper sample size, which represents more OiP in a surgical ward than those found in many other papers. In addition, several nutritional anthropometry indicators and nutritional screening were investigated. Another factor to be highlighted as a strong point of our investigation refers to the multiple linear regression that was applied with the purpose of identifying the variables associated with CC, but not for predicting this result, based on the assumption of cause and effect. Due to the transformation applied, the estimated parameters only served to direct the existing relationship and not for the calculation of the predicted values. Our study should be interpreted with some limitations, such as the fact that it was performed in a single center using a cross-sectional and retrospective design, which did not allow us to address other impacts. The most relevant aspects of this study are:

1. It was performed with 417 hospitalized patients in a surgical ward; however, it can be applied to other populations like those homebound and bedbound, unable to walk or stand, and therefore, unable to assess body weight.

2. The population herein is aged 65 years and older, male and female, with a high representation of older adults, which may benefit the current population of patients.

3. The CC indicator is easily applicable and can assist health professionals in assessing nutritional status of patients and risk of sarcopenia in the elderly. This article may be relevant to health professionals, and the topics that they are involved with and care about. This study suggests even greater attention by these professionals in treating the risk of sarcopenia in older adult patients. The CC is an effective and easily applicable measure in the hospital setting and may be better able to assess the nutritional status of OiP, especially in bedridden patients in a surgical ward.

## CONCLUSIONS

Gender, age, BMI, and AC were all together associated with CC, in addition to sga and absence of complications; and CC is a relevant indicator in clinical practice in OiP.
